# The Autophagy Status of Cancer Stem Cells in Gliobastoma Multiforme: From Cancer Promotion to Therapeutic Strategies

**DOI:** 10.3390/ijms20153824

**Published:** 2019-08-05

**Authors:** Larisa Ryskalin, Anderson Gaglione, Fiona Limanaqi, Francesca Biagioni, Pietro Familiari, Alessandro Frati, Vincenzo Esposito, Francesco Fornai

**Affiliations:** 1 Department of Translational Research and New Technologies in Medicine and Surgery, University of Pisa, via Roma 55, 56126 Pisa, Italy; 2 I.R.C.C.S. Neuromed, via Atinense 18, 86077 Pozzilli (IS), Italy; 3 Department of Neuroscience, Mental Health and Sense Organs NESMOS, Sapienza University of Rome, 00185 Roma, Italy

**Keywords:** rapamycin, glioma stem cells, stemness, differentiation, mTOR, Notch, Hedgehog, Wnt/β-catenin

## Abstract

Glioblastoma multiforme (GBM) is the most common and aggressive primary brain tumor featuring rapid cell proliferation, treatment resistance, and tumor relapse. This is largely due to the coexistence of heterogeneous tumor cell populations with different grades of differentiation, and in particular, to a small subset of tumor cells displaying stem cell-like properties. This is the case of glioma stem cells (GSCs), which possess a powerful self-renewal capacity, low differentiation, along with radio- and chemo-resistance. Molecular pathways that contribute to GBM stemness of GSCs include mTOR, Notch, Hedgehog, and Wnt/β-catenin. Remarkably, among the common biochemical effects that arise from alterations in these pathways, autophagy suppression may be key in promoting GSCs self-renewal, proliferation, and pluripotency maintenance. In fact, besides being a well-known downstream event of mTOR hyper-activation, autophagy downregulation is also bound to the effects of aberrantly activated Notch, Hedgehog, and Wnt/β-catenin pathways in GBM. As a major orchestrator of protein degradation and turnover, autophagy modulates proliferation and differentiation of normal neuronal stem cells (NSCs) as well as NSCs niche maintenance, while its failure may contribute to GSCs expansion and maintenance. Thus, in the present review we discuss the role of autophagy in GSCs metabolism and phenotype in relationship with dysregulations of a variety of NSCs controlling pathways, which may provide novel insights into GBM neurobiology.

## 1. Introduction

Gliomas are the most prevalent and lethal intracranial tumors in adults, accounting for approximately 36% of primary brain tumors [1]. Arising from glial cells, gliomas represent about 80% of malignant tumors of the central nervous system (CNS). Among these, astrocytomas including glioblastoma, constitute the largest subgroup.

Glioblastoma multiforme (GBM), a WHO grade IV malignant diffuse glioma, is the most frequent primary brain tumor in adults [2,3]. With a median overall survival of 14 months after diagnosis, this high-grade astrocytoma remains the most aggressive and lethal CNS tumor [4]. To date, conventional therapies provide only a slight improvement in the survival and quality of life of GBM patients [5]. Despite standard treatments with radiotherapy plus adjuvant chemotherapy, GBM frequently recurs with lower response rates to subsequent therapeutic approaches. Thus, advances in understanding GBM biology are urgently needed.

As suggested by the term “multiforme”, GBM is characterized by a marked intra-tumoral heterogeneity at both cellular and molecular levels. Among the multiple signaling pathways that have been implicated in GBM progression, the PTEN/PI3K/Akt/mTOR axis holds center stage being involved in tumor cell growth, proliferation, and metabolism [6]. The PTEN/PI3K/Akt/mTOR pathway is aberrantly activated in a variety of human cancers, including gliomas and GBM [7,8,9,10]. In detail, mutations in the tumor suppressor gene PTEN are reported in approximately 80% of GBM [11]. This leads to increased activation of the downstream effector molecule mTOR, which in turn sustains cell metabolism by promoting protein synthesis while suppressing autophagy, the major protein degradation pathway. A large body of clinical and experimental evidence indicates a key role of PI3K/Akt/mTOR hyper-activation in GBM biology [11,12,13,14,15,16,17]. An association between the PI3K/Akt/mTOR signaling and tumor malignancy is confirmed by studies documenting an upregulation of pAKT, pmTOR, and p-p70S6K in high-grade gliomas (grades III and IV) compared with low-grade gliomas (grades I and II) [18]. Again, the expression and phosphorylation of mTOR are associated with a worse prognosis in GBM [19]. In line with the evidence obtained in human GBM samples and cell lines, constitutive activation of mTOR in murine orthotopic xenograft models contributes to formation, growth, and progression of malignant glioma, thus recapitulating the main features of human GBM [12,20,21].

Among the various mechanisms through which PTEN/PI3K/Akt/mTOR hyper-activation sustains glioma cells metabolism, autophagy suppression plays a seminal role. As a proof of concept, the basal activity of autophagy is very low in astrocytomas including GBM from both patients and experimental models, while rescuing autophagy through mTOR inhibition is associated with a reduction of malignant glioma cells growth, proliferation, and invasion in vitro and in vivo [22,23,24,25,26,27,28,29,30,31,32].

Remarkably, mTOR hyper-activation and autophagy suppression are both implicated in a crucial standpoint of GBM pathophysiology, that is, maintaining the oncogenic properties of malignant glioma by promoting the growth and maintenance of glioma stem-like cells (GSCs). As it occurs in different hematopoietic and solid-tumors, GBM harbors a fraction of cancer stem cells known as GSCs, which are endowed with key features of normal neural stem cells (NSCs) of the adult brain, such as sustained self-renewal and proliferation [33]. GSCs are thought to be the driving force of GBM malignant phenotype since they are able to differentiate into phenotypically heterogeneous tumorigenic cancer cells and to establish and recapitulate a whole tumor upon intracranial transplantation [34]. Apart from contributing to GBM cellular heterogeneity, GSCs possess an increased therapeutic resistance, thus promoting tumor infiltration, treatment failure, and relapse [35,36,37,38,39].

mTOR and autophagy pathways are strongly related to the metabolism of both NSCs and GSCs [40,41,42,43]. The balance between mTOR activity and autophagy is seminal to modulate stem-cell niche homeostasis as well as quiescence, self-renewal, and differentiation of normal NSCs [44,45,46], while mTOR hyper-activation and autophagy impairment may occlude GSCs differentiation, thus sustaining the maintenance and expansion of the tumor stem cell niche [26,27,33,47,48,49]. Constitutive activation of the mTOR signaling and autophagy failure contribute to proliferation and pluripotency of GSCs [50,51,52,53,54]. On the other hand, mTOR inhibition and autophagy induction contribute to reducing stem cell-like properties, promoting differentiation, and restraining cell migration and invasion potential of GSCs [32,49,55,56]. In this scenario, dysregulations of mTOR and autophagy machinery do intermingle with a myriad of molecular pathways to sustain GSCs proliferation, GBM aggressiveness, and treatment resistance. This is the case of brain micro-environmental factors, which contribute to altering GSCs metabolism by acting on the mTOR pathway. Again, besides mTOR, autophagy is bound to several molecular pathways that regulate NSCs growth and differentiation and that are altered in GSCs and GBM. This is the case of Wnt/β-catenin, Notch, and Hedgehog pathways [57,58,59]. The present review aims to dissect those autophagy-related, mTOR-dependent and -independent biochemical pathways that characterize cancer stem cells in glioblastoma. Disclosing the mechanisms by which autophagy impairment may sustain GSCs self-renewal, proliferation, and resistance to therapies might provide novel insights into the neurobiology of GBM, and hopefully, contribute to the development of new therapeutic strategies.

## 2. Identification and Targeting of Cancer Stem Cells in Glioblastoma

The adult human brain possesses self-renewing and proliferative NSCs, which reside within restricted germinal regions, namely the subependymal ventricular zone (SVZ), the subgranular zone (SGZ) of the dentate gyrus of the hippocampus, and the subcortical white matter [60,61,62]. The SVZ of cornu temporalis of the lateral ventricle is one of the most active germinal regions within the adult human brain [63]. In fact, the SVZ continuously generates newborn differentiated neurons, thus being crucial in sustaining postnatal neurogenesis and maintaining the neurogenic niche of the mature brain. In the past 20 years, the identification of brain cancer stem cells (CSCs) as a therapeutic target has greatly improved the comprehension of the molecular pathways that are implicated in the pathophysiology of high-grade gliomas.

**Figure 1:**
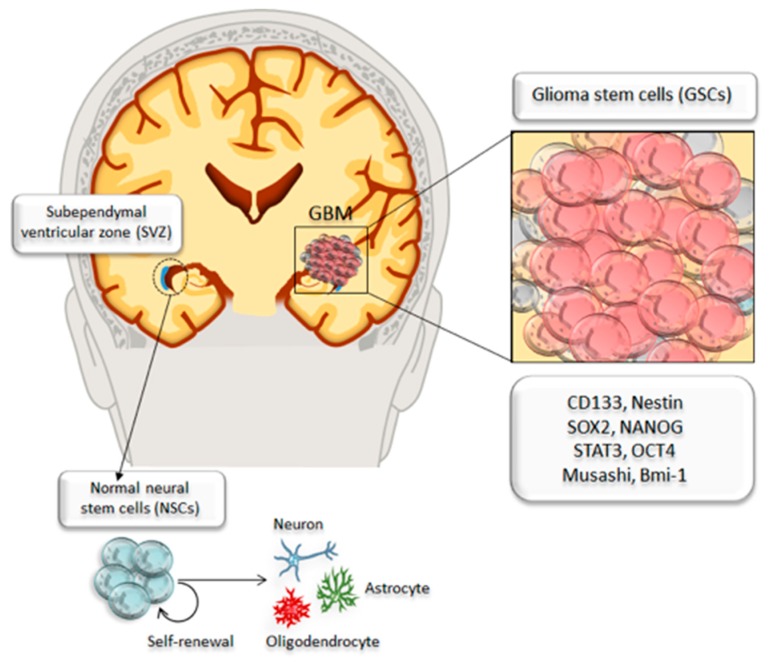
Identifying cancer stem cells in glioblastoma. Neural stem cells (NSCs) reside within the subependymal ventricular zone (SVZ), which represents the classic neurogenic niche of the adult brain. Within the SVZ, NSCs can undergo self-renewal or they can differentiate into neurons, astrocytes, and oligodendrocytes. The development of glioblastoma multiforme (GBM) depends on a small population of tumor cells known as glioma stem cells (GSCs). GSCs share several core properties of NSCs, such as stemness and sustained proliferation. GSCs that harbor tumor-initiating potential can be identified through specific markers such as CD133, Nestin, SOX2, NANOG, STAT3, Musashi, Bmi-1.

CSCs share core biological properties of normal NSCs, such as the potential of self-renewing and maintaining proliferation. In particular, GBM contains a subpopulation of CSCs with enhanced, long-term, self-renewal ability [33,34,64]. This is the case of GSCs residing in perivascular niches within the SVZ and the dentate gyrus of the hippocampus [65,66]. These cells potentially give rise to highly proliferative tumor cells, thus constituting a tumorigenic bulk within the healthy brain parenchyma (Figure 1). To date, it is still unclear whether GSCs originate from NSCs or undifferentiated neural/glial cells transform into CSCs; in any case, GSCs are considered to drive neoplastic transformation [67,68,69].

Fervent research has been carried out aimed at identifying specific subpopulations of GSCs that harbor tumor-initiating potential. Early in vivo studies demonstrated that a subpopulation of tumor cells expressing CD133 (Prominin-1) antigen was capable of tumor initiation when implanted into the adult NOD-SCID (non-obese diabetic, severe combined immunodeficient) mouse brains [34]. However, subsequent studies demonstrated that even CD133-negative glioma cells retain the ability to induce tumors in vivo [70,71]. Apart from the cell surface antigen CD133, which is classically associated with GSCs, these brain tumor cells populations express additional stem cell markers that are classically used to identify normal NSCs, such as Nestin, Bmi-1, and Musashi [72,73]. Other stem cells markers include CD15/SSEA-1, CD44, integrin α6, L1CAM, and A2B5 [74,75,76,77,78,79,80,81]. Moreover, several transcriptional factors are highly expressed in subgroups of GSCs, such as NANOG, SOX2, STAT3, OCT-4, and c-Myc [82,83,84,85]. These transcriptional factors regulate cancer stem cell properties, thereby contributing to CSCs self-renewal and pluripotency. Remarkably, their expression is under the control of mTOR via the activation of the PI3K/Akt pathway.

Nevertheless, none of these markers when considered alone is sufficient to confer stem cell-like properties to cancer cells. Again, due to the marked genetic and phenotypic heterogeneity, GSCs cannot be identified with a unique marker. Thus, the combined detection of different CSCs markers is needed to identify GSCs. For instance, Nestin, a type VI intermediate filament protein observed in NSCs, is frequently co-expressed along with other GSCs markers, such as CD133 or SOX2, and it is essential to confirm cell stemness [86,87].

Nonetheless, so far there is no specific biomarker being specific for the identification and isolation of CSCs in GBM. This stresses the need for a better understanding of the molecular mechanisms contributing to the invasive phenotype of GBM. Recent research points at mTOR signaling hyper-activation and subsequent autophagy suppression as key events implicated in GBM stem cell maintenance, tumor propagation, as well as treatment resistance, which will be dealt with in the following sections.

## 3. mTOR Function in Glioblastoma Cancer Stem Cells

The mammalian Target Of Rapamycin (mTOR) is a master regulator of cell growth, proliferation, and metabolism [88]. In particular, this kinase is a major effector of the PI3K/Akt pathway, which in turn is stimulated by several upstream environmental inputs, mainly nutrient availability and cellular energy levels. Upon ligands binding (i.e., growth factors, amino acids) to their respective transmembrane receptor, the upstream PI3K kinase phosphorylates Akt, which in turn activates the mTOR complex. Once activated, mTOR phosphorylates two major downstream effector molecules, namely p70S6K and 4E-BP1, which ultimately promote protein synthesis [88]. Whereas amino acids are conveyed through the PI3K/Akt/mTOR pathway to stimulate protein synthesis, they also inhibit autophagy, thus repressing protein degradation. In fact, insulin or nutrient-related signals activate mTOR, which in turn suppresses early steps in the biogenesis of autophagosomes by phosphorylating the ULK1 complex [89]. Conversely, nutrient depletion or administration of the lipophilic macrolide rapamycin inhibits mTOR activity, thereby stimulating the ULK1/ATG13/FIP200 complex formation, which is required to initiate autophagy [90]. Additionally, mTOR can indirectly control the autophagy pathway by inhibiting the transcription factor EB (TFEB), which regulates several lysosomal-related genes [91,92].

Over the last decade, aberrancies of mTOR signaling have been reported in several types of solid tumors, especially CNS tumors, where the constitutive hyper-activation of the PI3K/Akt/mTOR pathway represents one of the major contributors of tumor initiation and progression [16,93]. In particular, PI3K/Akt dysregulations may arise from different genetic alterations, encompassing mutations in upstream oncogenes and/or tumor suppressor genes, mutations in mTOR complex, or the mTOR gene itself [94,95].

In keeping with a role in cell proliferation, several studies demonstrated that mTOR is essential in CNS development and neural progenitor homeostasis. During brain development, mTOR dynamically regulates NSCs self-renewal and differentiation [63,96,97,98]. In fact, normal NSCs undergo self-renewing divisions to propagate the stem cell pool, but they also produce progenitor cells, which then differentiate into neurons, astrocytes, or oligodendrocytes. Proper activation of mTOR signaling is required for homeostatic regulation of normal NSCs niche. In baseline conditions, mTOR activity is finely tuned to maintain the delicate balance between proliferating and differentiating signals within the stem cell niche, where normal NSCs reside. There is a wealth of evidence relating dysregulated mTOR signaling with alterations in neural progenitor homeostasis [97,98,99,100,101,102]. For instance, mTOR hyper-activation in embryonic NSCs results in enhanced SVZ progeny generation and subsequent premature differentiation and impaired maturation [103]. At the same time, a persistent hyper-proliferation induced by mTOR hyper-activation may lead to the exhaustion of the NSCs pool. Such an effect can be reverted by the administration of the gold-standard mTOR inhibitor rapamycin [104]. Remarkably, the hyper-activation of mTOR signaling in transgenic mice results in a marked expansion of the SVZ stem cell compartment and subsequent glioma development [105]. Thus, it is not surprising that abnormal mTOR activity within NSCs is associated with severe CNS diseases ranging from brain tumors to neurodevelopmental disorders [106,107,108].

In keeping with GBM, mTOR has emerged as a critical cue in the maintenance of GSCs niche. Compared with normal NSCs, GSCs undergo uncontrolled proliferation and impaired differentiation, being a tumorigenic niche [78]. While finely tuned mTOR signaling is essential for normal CNS development, GSCs take advantage of an improper mTOR activity to fuel tumor growth and infiltration. This is also due to changes in the tumor niche micro-environment where GSCs receive proliferation signals that overcome growth-inhibiting ones. In fact, cell components within the brain tumor micro-environment, including endothelial cells, glia or neurons may promote GSCs proliferation by releasing signaling molecules such as mitogens, neurotrophic factors, and neurotransmitters.

It is remarkable that mTOR alterations are bound to several tumor-extrinsic mechanisms that support tumor growth and mediate GBM relapse and infiltration [109]. For instance, the activity-regulated secretion of the synaptic protein Neuroligin-3 (NLGN3) activates the PI3K/mTOR pathway to promote cell proliferation [110]. Similarly, neurotrophic factors released by astrocytes exert a proliferation-promoting effect on glioma cells [111]. In addition, it has been demonstrated that secreted factors released by endothelial cells reinforce GSCs stem-like phenotype through the mTOR pathway [51].

Hyper-activation of mTOR promotes self-renewal, proliferation, and pluripotency of GSCs, thus sustaining the oncogenic properties of malignant gliomas [50,52,53]. Conversely, the inhibition of mTOR with rapamycin counteracts these effects. For instance, the PI3K/Akt/mTOR pathway promotes the expression and activity of the transcription factor SOX2, which is required for the maintenance of GBM stem-like properties [112,113]. Conversely, rapamycin decreases GSCs self-renewal and proliferation through downregulation of SOX2 at both protein and mRNA levels [114]. Similarly, mTOR-dependent activation of the downstream effector HIF-1α enhances GSCs self-renewal and proliferation while maintaining their undifferentiated phenotype. In contrast, rapamycin abrogates these effects, which contributes to counteracting tumorigenesis [115]. Rapamycin also reduces GSCs sphere formation and the expression of GSCs-related markers, namely CD133 and Nestin [48]. This is in line with studies showing that targeting the PI3K/Akt/mTOR pathway represses stem-like cell properties in GBM cells by reducing the expression of other pluripotency-regulating transcription factors, such as NANOG and OCT-4 [116,117,118].

Similar results were documented by Mendiburu-Eliçabe et al. in two GBM patient-derived CSC lines, where rapamycin markedly reduces cell growth rate along with the stemness marker CD133 [119]. Moreover, Sunayama et al. [48] demonstrated that combined treatment with rapamycin and LY294002, a PI3K inhibitor, suppresses stemness while increasing the expression of the neuronal marker βIII-tubulin, which suggests a differentiating effect of dual PI3K/mTOR inhibition [48].

These data are in line with our previous studies demonstrating that rapamycin inhibits GBM cell growth in vitro and in vivo through gene expression changes which promote differentiation of GSCs towards a neuron-like phenotype [16,29,32]. Intriguingly, when administered in vivo to mice bearing GBM xenografts, rapamycin induces almost a total inhibition of tumor growth, which occurs in the absence of apoptotic or necrotic cell death [29]. Rapamycin counteracts GBM growth by suppressing the gene expression of the stemness marker Nestin, while stimulating those related to neuronal differentiation, encompassing both early and post-mitotic mitotic neuronal markers, such as βIII-tubulin, NeuroD, and NeuN [16,32]. Such an effect is associated with a reduced GSCs proliferation, tumorigenicity, and migration.

Recently, mTOR activation emerged as a crucial player in driving GSCs invasiveness, which represents another key factor contributing to GBM recurrence. In fact, hyper-activation of the Akt/mTOR pathway sustains GSCs migration and infiltration within the surrounding healthy brain parenchyma. As proof of concept, mTOR inhibitors suppress GSCs aggressiveness and invasive potential [117]. Moreover, mTOR inhibition downregulates both mRNA, protein levels, and the activity of the matrix metalloproteinases, MMP-9 and MMP-2, which promote tumor invasion through extracellular matrix degradation.

Overall, these findings suggest that mTOR inhibition coupled with radio- and/or chemo-therapy might hold great potential in hindering GBM progression.

## 4. Autophagy in Glioblastoma Cancer Stem Cells

It is widely recognized that autophagy is altered in GBM. This is not surprising since autophagy is essential in preserving stem cell homeostasis by finely tuning stem cell maintenance and differentiation [40,42]. On the other hand, altered autophagy may contribute to maintaining stem-like properties of GSCs, as well as diminished response to normal differentiation cues. Nonetheless, autophagy emerges as a double-edged sword in GBM development [120,121]. While in healthy cells autophagy acts as a tumor-suppressive mechanism by maintaining cell homeostasis, in cancer cells it may exert either a tumor-promoting or tumor-suppressing effect. Thus, it is still on debate whether autophagy induction or inhibition may represent the most promising approach for future GBM treatments. In addition, when attempting to analyze the autophagy status in GBM one has to face with multiple factors such as tumor stage, micro-environment, and GSCs heterogeneity.

Therefore, in the present section, we discuss the dual role of autophagy in GSCs generation, differentiation, migration/invasion, and treatment resistance, with a special emphasis on the emerging pro-differentiating effect of autophagy induction in GSCs. In fact, rather than extinguishing the GSCs population, current research has focused on forcing these cells to undergo differentiation through autophagy regulation.

### 4.1. Autophagy Promoting GSCs

A bulk of evidence points to the theory that autophagy inhibition may be beneficial when GBM cells are exposed to stressful stimuli, such as hypoxia, nutrient starvation, or even chemotherapy. In fact, a lack of oxygen and nutrients occur in GBM due to rapid tumor growth and insufficient nutrient supply from the lining vasculature, which may contribute to over-activating protective autophagy while desensitizing cells to chemotherapy. For instance, hypoxia increases the amount of CD133+ GSCs that are more resistant to BNIP3-dependent apoptosis compared to CD133 negative ones [122]. This may be due to BNIP3-dependent autophagy overactivation, which is associated with GSCs proliferation and chemoresistance [123,124]. Conversely, combined administration of autophagy inhibitors and chemotherapy drugs sensitizes GSCs to cytotoxicity [120,121,125].

Again, overactivation of BNIP3-dependent autophagy is also associated with an increased expression of MT1-MPP via JAK/STAT3 in GBM cell lines, which may contribute to the chemoresistant and invasive phenotype of GSCs [126].

Likewise, therapies targeting GSCs-promoting pathways such as Notch induce protective autophagy in glioma neurospheres, which is associated with chemoresistance since this latter is occluded by the combined treatment with autophagy inhibitors [127].

In this scenario, hypoxia-induced autophagy emerges as an adaptive mechanism that promotes cancer stem cell survival by ensuring nutrient and energy supply. In this context, tumor cells upregulate their levels of aerobic glycolysis while reducing mitochondrial energy supply. Thus, as the metabolic process rises, autophagy is recruited to support metabolic reconfiguration [128]. In fact, autophagy provides GCSs with necessary nutrients by recycling macromolecules and organelles. This same mechanism is thought to underlie autophagy-induced resistance to chemotherapeutic agents. In fact, autophagy can degrade dysfunctional organelles and reduce reactive oxygen species (ROS) accumulation, thus protecting the cell from pro-apoptotic stimuli while promoting genome stability. Such a cytoprotective mechanism may also result in the development of multidrug resistance (MDR).

From these studies, it emerges that autophagy inhibitors are most employed as a strategy to enhance chemotherapy-induced cytotoxicity. Nonetheless, recent studies suggest that drug-induced differentiation of GSCs may be a promising approach to eradicate GBM cancer stem cells. As we shall see in the next section, this is strongly bound to autophagy induction, which is key in controlling stem cells properties. As a proof of concept, activating autophagy inhibits GSCs proliferation, self-renewal, tumorigenesis, and reduces stemness, while restoring GSCs differentiation. This, in turn, may be beneficial in counteracting GBM progression.

### 4.2. Autophagy Combating GSCs

**Figure 2:**
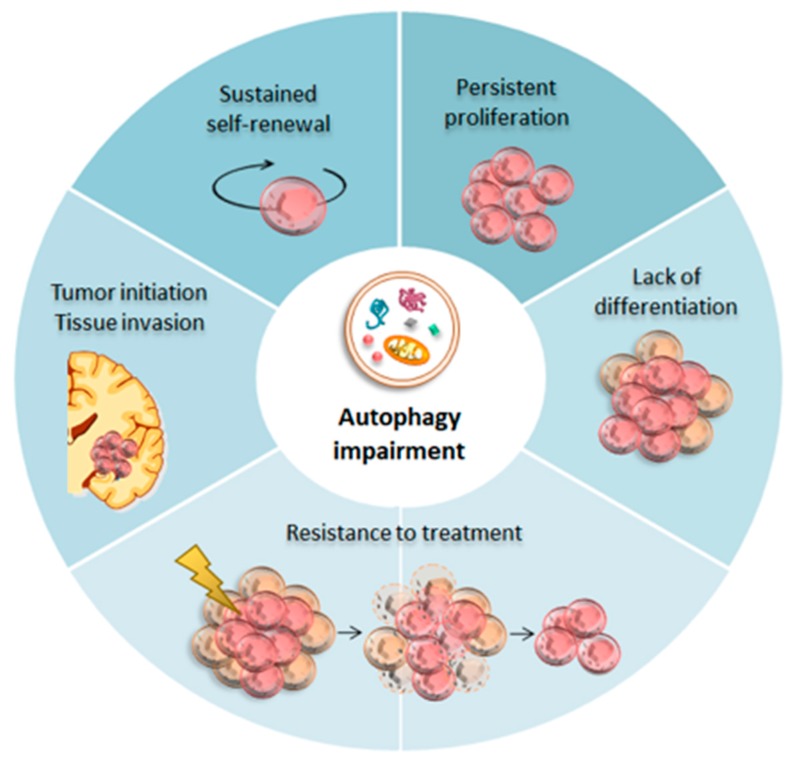
Autophagy in Glioma Stem Cells (GSCs). During development, baseline autophagy guarantees neuronal differentiation and homeostasis within the neural stem cells (NSCs) niche. Impaired autophagy seems to be crucial for GSCs tumor initiation. In fact, when autophagy is impaired (central while circle), GSCs undergo uncontrolled self-renewal and rapid proliferation in the absence of differentiation. GSCs also infiltrate within the healthy brain parenchyma, thus displaying enhanced invasion and resistance to therapy, the two hallmarks of GBM aggressiveness.

Autophagy induction, mainly through inhibition of the mTOR pathway, exerts anti-proliferative and pro-differentiating effects on glioblastoma stem-like cells (Figure 2). For instance, the antifungal agent itraconazole suppresses GSCs proliferation through induction of autophagy [30]. The in vitro anti-proliferative effect of itraconazole is confirmed in vivo using a subcutaneous GBM xenograft mouse model, where itraconazole significantly decreases GBM growth. Conversely, autophagy blockage, by knocking down either ATG5 or BECN1 with small interfering RNA (siRNA), reverts the anti-proliferative effect of itraconazole. In detail, itraconazole acts as an autophagy inducer by downregulating the sterol carrier protein 2 (SCP2), which decreases cholesterol trafficking towards the plasma membrane to inhibit of the Akt/mTOR pathway [129].

Enhancing autophagy in GSCs produces a variety of effects well beyond the mere inhibition of cell proliferation (Figure 2). In fact, defective autophagy has been implicated in the maintenance of the oncogenic properties of GSCs, such as stem-like properties, self-renewal ability, and pluripotency. Conversely, autophagy induction through mTOR inhibition suppresses GSCs self-renewal and tumorigenicity in vitro and in vivo through promoting autophagy-dependent degradation and inhibition of Notch1 [43]. In fact, upregulation of Notch1 sustains glioma stem cell phenotype, while autophagy induction counteracts such an effect by suppressing Notch1 signaling [43]. Again, metformin-induced Akt/mTOR inhibition impairs GSCs sphere formation, an indirect index of self-renewal, via autophagy induction [130].

Again, autophagy induction suppresses GSCs aggressive phenotype through promoting GSCs differentiation towards a neuron-like phenotype [27,32,131,132]. A failure of autophagy machinery also contributes to GSCs chemo- and radio-resistance [133,134]. Remarkably, rapamycin-induced autophagy prevents GSCs chemo-resistance, while promoting cell differentiation [27]. Likewise, cannabidiol promotes GSCs differentiation through autophagy-dependent upregulation of Aml-1 transcription factors, which is associated with reduced GSCs chemoresistance, proliferation, and clonogenic potential [135]. This is in line with studies showing that inhibition of miR-17, which activates autophagy gene expression, suppresses tumor progression and improves chemo-and radio-therapy [136].

Autophagy is crucial for GSCs invasiveness. In fact, rapamycin-induced autophagy impairs GBM cell migration [32,55]. Stimulation of autophagy reverts GCSs invasiveness by reducing two transcriptional factors belonging to SNAI family, which control epithelial–mesenchymal transition (EMT). Conversely, when autophagy is occluded via silencing Atg5 and Atg7 GBM cell migration and invasion are enhanced [55].

Micro-environmental factors may influence GSCs phenotyope by affecting the autophagy machinery. For instance, CXCR4 ligands favor GSCs chemotactic migration through inhibition of autophagy. In fact, abnormal stimulation of CXCR4 triggers a marked reduction in autophagosomes biogenesis in GBM cancer cell lines while favoring the formation of adhesion complexes to the extracellular matrix [137]. Again, mitogen deprivation was shown to inhibit GSCs self-renewal and survival via the engagement of mTOR-dependent autophagy. On the other hand, blocking autophagy reproduces the effects of ex-vivo administered endothelial-secreted factors, that is promoting GSCs survival and stemness [51].

mTOR-dependent autophagy activation associates with the anti-proliferative effects that are induced by silencing CD164 (endolyn), a key factor implicated in GBM growth [138]. In detail, CD164 is a member of sialomucin family, which plays a role in proliferation, adhesion, and differentiation of hematopoietic stem cells [139] and different types of tumors, including gliomas [140]. When over-expressed, CD164 activates the PI3K/Akt/mTOR pathway to sustain GBM growth [140]. On the other hand, CD164 downregulation reduces glioma cell proliferation, migration, and tumor invasion via depression of the Akt/mTOR pathway and autophagy induction [138].

In recent years, natural compounds besides rapamycin, such as curcumin and resveratrol were used in GBM research owing to their anti-proliferative, anti-migratory, and anti-invasive effects on GSCs [27,116,130,141,142,143]. Most of the beneficial effects of these compounds are due to autophagy stimulation via PI3K/Akt/mTOR pathway inhibition. For instance, treatment with curcumin suppresses GSCs self-renewal and proliferation in vitro and in vivo while inducing GSCs differentiation through activation of the autophagy pathway [131]. In detail, curcumin-treated GSCs feature an up-regulation of the differentiation markers Tuj1, GFAP, Olig2, and βIII-tubulin, and a concomitant downregulation of the stem-like markers CD133 and Nestin. Such a phenotypic switch induced by curcumin is accompanied by a marked stimulation of autophagy in both GSCs cultures and xenograft tumors. This was evidenced by an increase in LC3 immunofluorescent puncta, LC3 immunoblotting, and ultrastructure of autophagosomes. Recent in vitro studies demonstrate that curcumin-induced autophagy suppresses GSCs migration and invasion [143].

Recently, resveratrol, a natural polyphenolic antioxidant found in grapes and red wine, has shown beneficial effects against malignant glioma cells [116]. Growing evidence suggests that the anti-tumor effects of resveratrol consist in a reduction of GSCs proliferation and self-renewal along with promoting GSCs differentiation [144]. Thus, resveratrol contributes to the depletion of GSCs niche and suppression of tumor growth. It is remarkable that these effects are all accompanied by enhanced autophagy, as confirmed by the upregulation of the autophagy proteins Atg5, Beclin-1, and LC3-II and the induction of autophagosome formation [144,145]. Again, similar to curcumin, resveratrol-induced autophagy can restrain the invasive behavior of malignant glioma cells by suppressing GSCs adhesion and migration [142].

Likewise, berberine, an isoquinoline alkaloid isolated from *Berberis vulgaris* L., enhances autophagy flux in GSCs cells through the inhibition of the AMPK/mTOR/ULK1 pathway. Remarkably, this effect associates with a reduction in the proliferative potential and invasive properties of GBM cells [146].

Again, nigericin, a polyether antibiotic derived from *S. hygroscopicus* that affects mitochondrial ion transport, was shown to suppresses the proliferation of GBM cells along with the inhibition of GSCs stem-like properties, which associates with marked induction of autophagy [147].

## 5. The Cross-Talk between Autophagy and Glioblastoma Stem Cells-Controlling Pathways

Apart from the PI3K/Akt/mTOR pathway, autophagy machinery interacts with many proteins and signaling pathways that are implicated in GBM stem-cell properties. These include Wnt/β-catenin, Hedgehog, Notch, Histone deacetylases (HDAC), STAT3, and the de-ubiquitinase ubiquitin carboxyl-terminal esterase L1 (UCHL1). Indeed, rather than acting independently in sustaining GSCs growth and proliferation, these pathways merge to produce a chain of epigenetic, transcriptional, metabolic, and post-translational events where autophagy plays a central role.

### 5.1. Wnt/β-Catenin, Notch, and Autophagy in GSCs

When Wnt/β-catenin and Notch pathways are aberrantly activated GSCs self-renewal, proliferation, and invasion occurs [148,149,150]. On the other hand, either single or dual inhibition of Wnt/β-catenin and Notch signaling promotes GSCs neuronal differentiation, inhibits their clonogenic potential, decreases radio-resistance and halts tumor growth [148,149,150]. Remarkably, these effects are reproduced by autophagy activators since downregulation of both Notch and Wnt/β-catenin in GBM cells relies on the very same autophagy pathway [43,151,152]. In fact, autophagy activation is seminal to degrade Notch1 and Dishevelled, an activator of Wnt/β-catenin. Autophagy also re-locates β-catenin within the cell by moving the nuclear protein towards the plasma membrane where it associates with N-cadherin to form epithelial-like cell-cell adhesion structures [152]. This is in line with an increase N-cadherins and induction of a molecular switch from a mesenchymal to an epithelial-like phenotype in GBM cellular models upon autophagy stimulation [55].

### 5.2. UCHL1 and Autophagy in GSCs

UCHL1 de-ubiquitinase is up-regulated in several cancers, including pediatric high-grade gliomas, where it contributes to promoting GSCs self-renewal, transformation, and invasion [153]. The activity of UCHL1 is linked to dysregulations of Akt, mTOR, and Wnt/β-catenin pathways [154,155,156,157] and, remarkably, autophagy suppression [158,159]. For instance, UCHL1 activates Wnt signaling through de-ubiquitination and stabilization of β-catenin [160]. Likewise, UCHL1 enhances mTORC2 stability, thus activating Akt signaling [157]. Aberrant activation of UCHL1 suppresses autophagy either by interacting with LC3 or by inducing PDGFB (platelet-derived growth factor B)-dependent mTOR phosphorylation [158,159]. Silencing UCHL1 in patient-derived glioma cells is associated with decreased GSCs self-renewal, proliferation, and invasion [153]. Remarkably these effects occur along with a 70% reduction in Wnt signaling, and again, PDGFB ranks among the top upstream regulators of the effects induced by UCHL1 silencing [153], suggesting that autophagy may be involved in the anti-proliferative effects of UCHL1 inhibition in GSCs.

### 5.3. SOX3, Hedgehog, and Autophagy in GSCs

SOX3 is remarkably increased in primary GBM, where it is suggested to promote the malignant behavior of GSCs by enhancing their self-renewal, proliferation, viability, migration, and invasion [161]. SOX3 up-regulation in GBM cells is accompanied by an enhanced activity of the Hedgehog signaling pathway and remarkably, by suppression of autophagy [161]. This is not surprising since a cross-talk exists between Hedgehog and autophagy pathway [162], and dysregulations of one pathway may affect the other to converge in GBM tumorigenesis and GSCs maintenance. For instance, mTOR hyper-activation enhances the expression Hedgehog pathway while amplifying its target genes to promote GSCs regeneration, proliferation, and invasion [163]. On the other hand, the combined inhibition of PI3K/Akt/mTOR and Hedgehog pathways is more effective in suppressing GBM growth, GSCs self-renewal, proliferation, and EMT compared with single pathway inhibition.

### 5.4. STAT3 and Autophagy in GSCs

Enhanced STAT3 phosphorylation, which is required for GSCs proliferation and maintenance of multi-potency [83], is associated with Notch hyper-activation [164] and autophagy down-regulation in GBM cells [134,145,165]. In fact, activation of JAK2/STAT3 signaling pathway by HMGB-1 (High mobility group box 1) leads to autophagy inhibition [166], while administration of autophagy activators in GBM models produces a concomitant STAT3 inhibition, associated with increased GSCs chemo-sensitization, decreased self-renewal and proliferation [134,145,165].

### 5.5. Epigenetic Enzymes and Autophagy in GSCs

Recently, aberrant expression and activity of HDACs have been implicated in GBM onset and progression [167]. HDAC inhibition induces GBM cell growth arrest in orthotopic xeno-transplanted mice, and it reduces neurosphere formation from patient-derived GSCs while inducing a neuronal-like phenotype as evident by Tuj-1 upregulation [167]. Remarkably, these effects are promoted by autophagy, while autophagy inhibition counteracts the pro-differentiating effect of GSCs, which is induced by HDAC inhibition [167].

A similar effect is obtained when autophagy is pharmacologically inhibited in GSCs that are treated with a G9a histone methyltransferase inhibitor (BIX01294) [168]. In detail, in glioma cell lines and GBM derived cell cultures, aberrant expression of a G9a histone methyltransferase is bound to low expression levels of both autophagy and differentiation-related genes. Thus, administration of BIX01294 promotes autophagy-dependent GSCs differentiation [168].

**Figure 3:**
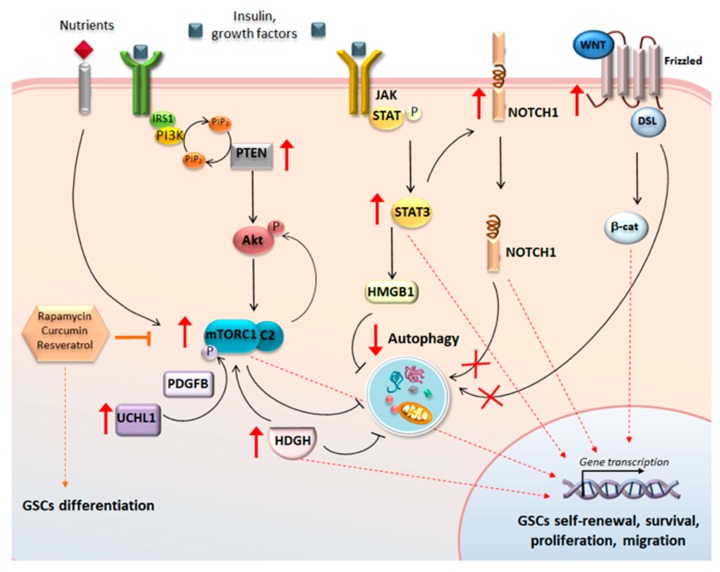
Molecular pathways at the crossroad between autophagy and glioma stem cells proliferation. An interdependency exists between autophagy and the foremost molecular pathways that contribute to GBM features by increasing stemness. For instance, hyperactive mTOR due to either nutrient/growth factor abundance, PTEN mutations, or UCKH1-related PDGFB-dependent phosphorylation of mTORC1, leads to autophagy suppression and mTOR-related transcriptional changes in the nucleus. UCHL1 also enhances mTORC2 stability thus potentiating activation of Akt signaling and mTOR hyperactivation. Again Hedgehog (HDGH) upregulation contributes to activating mTOR, suppressing autophagy and translocating HDGH within the nucleus. Likewise, JAK/STAT signaling leads to upregulation of STAT3, which impairs autophagy through HMGB. STAT3 may also contribute to Notch upregulation, which produces persistent transcriptional changes in the nucleus since its degradation is occluded when autophagy is impaired. Similarly, sustained Wnt/β-catenin activation occurs since the autophagy-dependent degradation of Dishevelled (DSL) is impaired. Altogether these events converge in transcriptional changes, which enhance GSCs self-renewal, proliferation, multi-potency, and migration. Rescuing autophagy through mTOR inhibitors such as rapamycin, curcumin, and resveratrol reverts these biochemical events while promoting GSCs differentiation.

The present findings provide evidence about a key role of autophagy in GBM in the light of the interplay with a plethora of intracellular signaling pathways which sustain GSCs malignant phenotype (Figure 3).

## 6. Conclusions and Future Perspective

The evidence here discussed converges in that autophagy plays a crucial role in GSCs phenotype and GBM malignancy. However, much remains to be elucidated in terms of molecular mechanisms that mediate mTOR dependent and independent autophagy, which remains an important topic of investigation in glioma. Apart from mTOR, a variety of molecular pathways are implicated in GSCs neurobiology and GBM aggressiveness such as STAT3, Wnt/β-catenin and Notch signaling. All these are interconnected to mutually enhance autophagy impairment. Nonetheless, it is worth mentioning that controversial results on autophagy status in GBM still exist in the literature. In fact, some studies report that enhanced autophagy may be implicated in tumor progression. On the one hand, this has to face with the fact that a variety of factors such as tumor micro-environment and tumor stages may differently impact on the autophagy status and flux. On the other hand, misinterpretations often occur when assessing the autophagy status. This is best exemplified by an increase in LC3 levels, which does not necessarily reflect increased autophagy, since it may be due to an accumulation of autophagosomes that do not fuse with lysosomes. Thus, the assessment of autophagy status depends on a careful evaluation of several autophagy markers, such as LC3-I/II ratio, subcellular localization, Atg12–Atg15 accumulation, and p62 degradation along with an assessment of the autophagy flux [169].

In summary, considering the neurobiology of GSCs, the role of autophagy is even amplified compared with the well-established effects already evidenced on GBM as a whole. In fact, autophagy strongly impacts GSCs maintenance, proliferation and resistance to therapies.

## Abbreviations

Aml-1	Acute myeloid leukemia 1
BNIP3	BCL2/adenovirus E1B 19-kDa protein-interacting protein 3
CNS	Central nervous system
CSCs	Cancer stem cells
EMT	Epithelial-mesenchymal transition
GSCs	Glioma stem-like cells
HDAC	Histone Deacetylases
mTOR	mammalian Target of Rapamycin
MDR	Multidrug resistance
MT1-MMP	Membrane Type-1 Matrix Metalloproteinase
NLGN3	Neuroligin-3
NSCs	Neural stem cells
PDGFB	Platelet-derived growth factor B
PI3K	Phosphoinositide 3-kinase
ROS	Reactive oxygen species (ROS)
SCP2	Sterol carrier protein 2
SGZ	Sugranular zone
SVZ	Subependymal ventricular zone
TFEB	Transcription factor EB
UCHL1	ubiquitin carboxyl-terminal esterase L1
